# Enhancing Energy Saving in Smart Farming through Aggregation and Partition Aware IOT Routing Protocol †

**DOI:** 10.3390/s20102760

**Published:** 2020-05-12

**Authors:** Karim Fathallah, Mohamed Amine Abid, Nejib Ben Hadj-Alouane

**Affiliations:** 1 National Engineering School of Tunis, University of Tunis El Manar, 1002 Tunis, Tunisia; nejib_bha@yahoo.com; 2 Fakultät für Informatik und Mathematik, University of Passau, 94032 Passau, Germany; Amine.Abid@uni-passau.de

**Keywords:** RPL, PA-RPL, WSN, 6LoWPAN, Smart Farming, In-network data aggregation, IoT

## Abstract

Internet of things (IoT) for precision agriculture or Smart Farming (SF) is an emerging area of application. It consists essentially of deploying wireless sensor networks (WSNs), composed of IP-enabled sensor nodes, in a partitioned farmland area. When the surface, diversity, and complexity of the farm increases, the number of sensing nodes increases, generating heavy exchange of data and messages, and thus leading to network congestion, radio interference, and high energy consumption. In this work, we propose a novel routing algorithm extending the well known IPv6 Routing Protocol for Low power and Lossy Networks (RPL), the standard routing protocol used for IPv6 over Low-Power Wireless Personal Area Networks (6LoWPAN). It is referred to as the Partition Aware-RPL (PA-RPL) and improves the performance of the standard RPL. In contrast to RPL, the proposed technique builds a routing topology enabling efficient in-network data aggregation, hence dramatically reducing data traffic through the network. Performance analysis of a typical/realistic precision agriculture case, considering the potato pest prevention from the well-known late blight disease, shows that PA-RPL improves energy saving up to 40% compared to standard RPL.

## 1. Introduction

The Internet of things (IoT) [1] is a networking paradigm that covers the extension of the traditional Internet to integrate the so called Lossy and Low power Networks (LLNs). A LLN is mainly made up of a high number of tiny and low cost IP-enabled sensor nodes with limited power, limited memory, and limited processing resources. They are interconnected using a variety of short range, low rate, lossy, and low power communication protocols. Since its advent, IoT enabled the emergence of various novel applications including smart buildings, smart cities, smart grids, etc. In this work, we rather focus on a particular an interesting application domain of IoT, that is precision agriculture (PA), also referred to as Smart Farming (SF) [2].

When an LLN is deployed in a large area, it is mandatory to use a routing protocol in order to route/guide data through multi-hop connections [3]. This was the main goal of the Routing Over Low power and Lossy networks (ROLL), originally defined by the Internet Engineering Task Force (IETF) in [4]. In March 2012, this group proposed a new routing protocol, called Routing Protocol for Low power and lossy networks (RPL) [5], designed for IP-enabled smart objects [6].

RPL [5] has quickly gained in popularity, thanks to various factors; most importantly, its flexibility to adapt to different network topologies [7]. While RPL was developed to serve four main applications at first: home automation, industrial control, urban environment monitoring, and building automation. Therefore, and by extending the use of RPL in different other domains, it is expected that any new application, Smart Farming as an example, will bring new issues, constraints, and challenges.

For instance, a farm is generally composed of a set of parcels. Each parcel is a contiguous piece of land in which one single crop is cultivated [8]. The partition of the land into parcels is a well known farming management technique in arable spaces. In fact, this spatial partitioning approach of the agricultural field into parcels simplifies the management of the farming activities (irrigation, fertilization, etc.,). In addition, partitioning enables the farmer to diversify his crops both in space and time, either by cultivating several crops in his land, or by growing one single type of crops at different rates insuring a continuous production over time.

Moreover, a farm may include other specialized buildings/facilities. These facilities could be dedicated either for breeding animals (stable, sheepfold, piggery, hen-house, hutch, breeding battery, and dovecote), cultivating special plantations (greenhouses, aquaponic/hydroponic farms, or tunnels), storing earth products (dryer, hayloft, barn, attic, silo, cellar), preserving high value materials (shed, hangar, ironwork, hut), or processing/transforming basic products (cereals, wine, cider, dairy, cheese, oil-seeds, etc.).

Within the Smart Farming context, each of these special buildings and parcels requires surveillance and monitoring of a set of critical parameters [9]. They should be first collected then forwarded to a decision support system (DSS) [10]. Based on the collected data, the DSS gives insights to the farmer about how to appropriately face issues occurring in each parcel (or sector). This aims to support the whole farming activities including irrigation, pest protection, fertilization, infrastructure control, automated harvesting, alarms management, feed distribution, etc. It is important to note that each farm sector (parcel or building) usually has a built-in infrastructure [11], such as drip irrigation system, air conditioning settings, complex machinery, etc.

Straightforwardly, and because of its intrinsic complexity, it is clear that a smart farm [12] will require an appropriate/accurate monitoring of a high number of critical parameters; in addition to the control of an important number of actuators [9]. Hence, when the surface, the diversity, and the complexity of the farm increase, the number of sensing nodes is expected to increase proportionally. However, as the number of nodes in the LLN increases, the volume of data similarly increases as well; leading to problems like network overhead, traffic congestion, bandwidth consumption, transmission delay, etc.

Inherently, in this application domain, in-network data aggregation techniques become naturally very attractive; mainly because of their capacity to reduce the amount of transferred data [13]. However, and due to the typical context of a smart farm [12] in terms of data heterogeneity and parcel/sector level aggregation requests, standard RPL clearly turns out to be inefficient for the implementation of many kinds of in-network aggregation techniques. Through the paper, we illustrate the RPL limitations, especially in terms of in-network aggregations.

This motivates the need for an improved RPL, which is capable of building a routing topology that enables efficient in-network data aggregation, taking into account the physical partition of the farm into parcels/sectors. For this purpose, we propose a new objective function to be integrated into RPL [5], so that the built tree-like structure rooted at the sink node, called Destination Oriented Directed Acyclic Graph (DODAG), becomes more suitable and more appropriate for such type of applications. Our proposal is an enhanced version of RPL, and is called PA-RPL, standing for Partition-Aware RPL [14].

An attractive feature of this proposal is the fact that its tree structure reflects the spatial partition of the monitored farm. With such a structure, the collected data in each parcel will be gathered in one particular elected node within the parcel, called Parcel Head (PH), in order to be processed/aggregated before being sent upward to the sink node. This approach is intended to improve RPL in order to enable efficient in-network data aggregation of the collected information for the monitoring/decision system in SF.

To the best of our knowledge, none of the literature research works paid enough attention to optimizing the DODAG structure through considering the spatial partitioning of farmlands. In this paper, we recall our proposal, PA-RPL [14], and show how it can be used to enable in-network processing leading to better performance. The key contributions of this research are summarized as follows:

1. We first recall our new RPL-based routing algorithm, called PA-RPL [14], that adapts the structure of the DODAG, considering the spatial division of the monitored farmland. Through its inherited nature, we show how our proposal enables efficient implementation of aggregation techniques applied to the upward traffic.
2. We then present a proof of concept of our proposal through the simulation of an arbitrary farmland composed of a variable number of parcels.
3. We also assess the performance of PA-RPL [14] in comparison to standard RPL, in case of a centralized data collection scenario, and when high percentage of parcels are simultaneously monitored. We show that in both cases, PA-RPL presents good performance comparable to those of the standard RPL. In contrast, when only few parcels are simultaneously supervised, the PA-RPL shows high gain in energy, reaching up to 20%.
4. Finally, we demonstrate the superiority of PA-RPL over the standard RPL through simulating a realistic Smart Farming application. In this work, we have considered a particular application aiming to detect and prevent the dangerous potato phytophora infestants disease. Our simulation and analysis report substantial energy savings that go up to 40%.

The remainder of this paper is organized as follow. In Section 2, we report a set of related works that concern the application of RPL in the context of farming. In Section 3, we describe the mechanism of standard RPL, while in Section 4, we present our proposal, the PA-RPL algorithm [14]. Section 5 is devoted to present the usefulness of In-network aggregation in the context of precision agriculture and the ability of PA-RPL to easily integrate such a mechanism compared to the standard RPL. The performance evaluation of PA-RPL and its comparison to the standard RPL through a case study, will be the object of Section 6. In this section, we evaluate the performance of PA-RPL in a realistic application that considers potato farm pest prevention. Section 7 serves to discuss the main advantages of the PA-RPL and its potentials. Finally, Section 8 concludes the paper.

## 2. Related Works

Even though that routing requirements in Smart Farming were not explicitly considered by RPL, recent studies show the important and promising adoption efforts of the IoT in agriculture [2,15,16,17]. In fact, the first advantage of IoT is to enable Machine-to-Machine (M2M) communications, between all farming infrastructure components. This provides farmers an advanced automation level, enabling the supervision and control of farm sectors with minimum farm–worker intervention. Indeed, in this extremely heterogeneous context, RPL enables interoperability among network components [18]. This interoperability, between densely deployed smart farm IP-enabled sensor/actuator nodes, generates substantial data exchanges that should be routed by RPL. Recall that RPL was mainly conceived for low data rate network. As a result of this, it is reasonable to state that one way to enable RPL [5] to be efficiently scalable in smart farms, is to find a way to reduce the routed data streams.

In the literature, recent research contributions considered applying the RPL protocol in the context of SF. In [19], the authors defined a scalable context-aware objective function (SCAOF) that can adapt RPL to the monitoring of a smart farm. This combined the awareness of energy, reliability, and robustness according to a composite routing metric approach. In [20] authors proposed agriculture data aggregation scheme for low-power and lossy SF networks. They first use the RPL routing protocol in agricultural system, especially in greenhouses. Then, they collect and analyze the sensed data before transmission up to the sink. In [21], the authors predicted the optimum values of the DODAG Information Object (DIO) interval rate and redundancy frequency that minimizes the battery consumption. In [22], the authors explored how to apply RPL for smart irrigation system. They identified the scalability as a main concern of RPL in smart farming context because of the high number and density of nodes, through the farm.

Most of the aforementioned works have focused on various aspects such as reducing the power consumption (using a related metric in the OF), bounding the delay, fostering the use of efficient links, etc. However, only few of them have paid enough attention to the optimization of the built routing structure, i.e., the DODAG, serving as the backbone infrastructure for any in-network aggregation we can imagine. In this paper, we focus on enhancing RPL in the SF context, by proposing a new objective function that optimizes the generated routing DODAG, so that it reflects the spatial partitioning of the monitored farm. Our proposal will be referred to as the Partition Aware-RPL (PA-RPL).

## 3. RPL Protocol Overview

RPL [5] is a distance vector protocol designed for IP-enabled smart objects. These entities are known to be highly constrained in terms of energy, memory, and computational capacity [6]. They form what we call Low power and Lossy Networks (LLNs). The LLNs are characterized by their dynamism, links’ unreliability, low data rates, and tolerance for packet loss.

**Figure 1:**
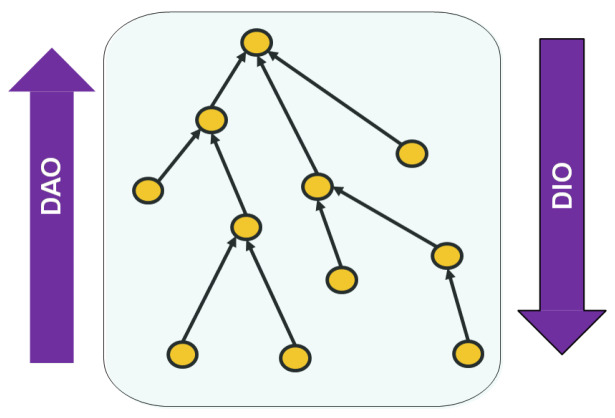
An example of RPL built routing topology (Destination Oriented Directed Acyclic Graph (DODAG)).

The main function of RPL [5] is to establish the organization/topology connecting the nodes in a network, ensuring cycle-free paths, between every node and the root (or sink). The obtained topology has a tree-like structure that is a Destination Oriented Directed Acyclic Graph, commonly referred to as DODAG (see Figure 1). It is worthy to note that the standard RPL, supports the creation and superposition of several DODAGs in the same network, each of which is rooted at a different sink node. For the sake of simplicity, we limit ourselves to one single sink node and a single DODAG.

The DODAG is gradually created through pro-actively sending DODAG Information Object (DIO) messages. The root node, i.e., the sink, initiates the building process. It sends the first DIO message, allowing its immediate neighbors (potential children), to join the DODAG by selecting the root as a parent. A second wave of DIO messages is generated by the already joined neighbors to invite their own immediate neighboring nodes (new potential children) to also join the DODAG: as such, former children become parents and the original root becomes grand parent. Consequently, a node could receive more than one DIO coming from higher layer neighbors. It selects a preferred parent based on an objective function (OF).

To avoid loops formation, a node maintains a Rank reflecting its depth relatively to the root. This particular information is calculated using the Rank of the preferred parent, to which a node adds a positive quantity, qualifying the wireless link, linking both of them (i.e., 1 if the rank represents the number of hops to the root [23], or more generally, the local Expected Transmission Count (ETX) [24] value if the Rank that represents the path ETX value to the root [25], etc.).

Hence, the Rank of a sensor needs to be carried over to its neighborhood by its dissemination in the DIO messages. Upon its reception, a neighbor node updates its own information if it finds that its own Rank is larger than the one included in the received message (i.e., the one used by the parent node).

The built DODAG is used for routing purposes. In fact, RPL defines two directions for communication: UP and DOWN. While the first refers to the direction “leaf sensor nodes → DODAG root”, the second refers to the opposite direction. In both directions, data-flows travel using the DODAG edges either UP or DOWN. With such routing structure, RPL supports three types of communication: (i) Many-to-One (i.e., Multi-point to Point: MP2P), representing a communication in the UP direction. (ii) One-to-Many (i.e., Point to Multi-point: P2MP), representing a communication in the DOWN direction. (iii) One-to-One (i.e., Point to Point: P2P), representing a communication in the either directions or in both simultaneously: from a node to another, thus we may need to go UP, and then DOWN to reach the destination.

It is worth mentioning that besides the DODAG Information Object (DIO) messages, RPL defines two additional types of messages. The first is called the DODAG Information Solicitation (DIS) message, which is periodically sent by a node, to solicit from its neighborhood any information regarding the formation of a DODAG. The second is called the DODAG Destination Advertisement Object (DAO) used to enable downward traffic. For this, a node advertises a so-called “prefix reachability towards the leaf nodes of a DODAG”.

## 4. Partition Aware-RPL Protocol Overview

In this section, we present our proposed algorithm, called PA-RPL, which stands for Partition Aware-RPL [14]. In order to emphasize the difference of PA-RPL with the standard RPL, we consider a typical smart farm, equipped with a set of sensors/actuators, uniformly distributed over its surface, aiming at ensuring its monitoring. At first, we show the DODAG built by the application of the standard RPL. We then, move to our proposal, PA-RPL, and show how it differs from the standard RPL.

**Figure 2:**
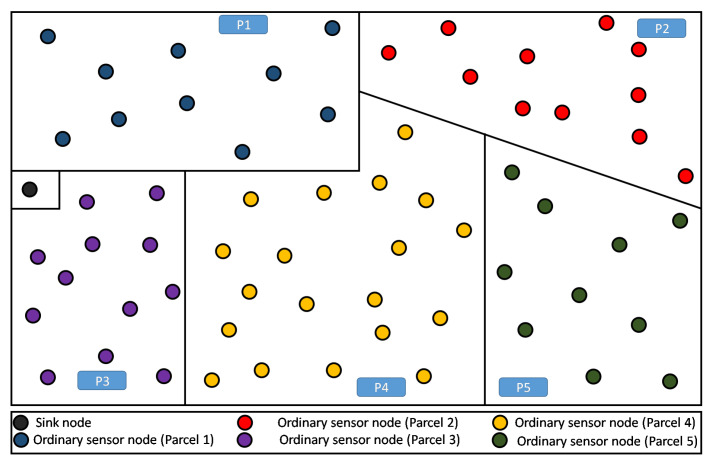
A farmland partitioning example: 5 parcels with randomly spread nodes.

Our considered farmland is rectangular (see Figure 2), where one or more types of crops are implanted. Note that the rectangular shape of the farmland represents an arbitrary choice, corresponding to a use-case scenario. Thus, the present discussion remains valid for any different shape without loss of generality. This land is divided into several parcels, each of which is intended to hold simultaneously a single or different kinds of crop (i.e., tomatoes, potatoes, carrots, etc.).

Throughout the farmland, IP-enabled wireless sensor/actuator nodes are uniformly distributed to monitor the cultivation. This includes the collection of data (such as the temperature or soil moisture), and the control of the irrigation and fertilization processes, etc. The start-up of the PA-RPL protocol follows almost the same steps as that of the RPL protocol. However, in PA-RPL it is necessary to pre-configure each node in order to make it know its position/adherence to a particular parcel. Information about the specific node partition can be obtained and/or simply configured in the node during deployment. This configuration can be easily done at the time of deployment by inserting in the flash memory of each node a small file containing the location of the node. Alternatively, over-the-air programming techniques can be used to provide each node with this information after deployment. A third option would be to disseminate the information in the network by flooding: the sink node floods such information in the network at the very beginning of the deployment. Each node receiving a message about itself keeps the position/color information and use it to build the DODAG as described in the rest of this section.

In our simulation, once a node knows its partition (or parcel), it is assigned a color that is specific for its parcel. In the example of Figure 2, there are five parcels having five respective colors for their respective nodes. The sink is assigned a sixth color that is different from the others, avoiding considering it as a member of any particular parcel.

**Figure 3:**
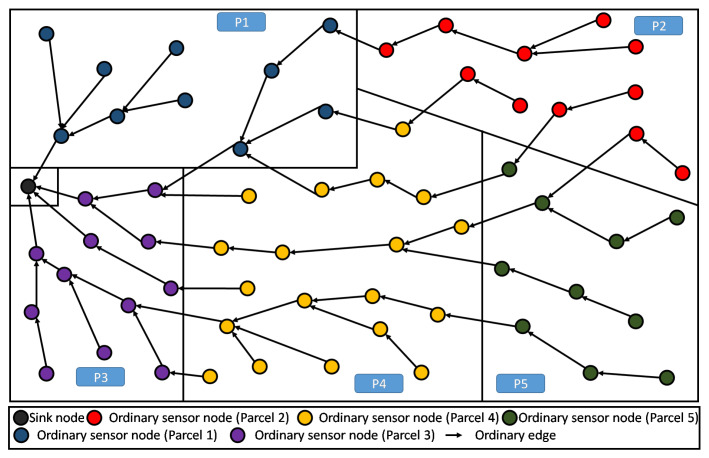
DODAG resulting from standard RPL deployed in a sample farmland.

The application of RPL would result in a structure similar to the one depicted in Figure 3. Recall that this DODAG is incrementally created/built based on an objective function (OF) to be satisfied. For the standard RPL, the physical land partitioning, according to crops, is totally transparent to the used OF, and thus, the built DODAG is completely independent from it. Of course, all communications are then driven by this tree-like structure. Adversely, PA-RPL aims at taking this physical partitioning into account when building the DODAG. This will be further discussed in the next two subsections.

### 4.1. Protocol Description

In order to be in conformity with the recommendations of RPL [5] standard, any customization (or improvement) of the initial routing protocol should be limited to the objective function (OF). In fact, the user can define his own OF depending on the application context and constraints. Note that the OF is the RPL mechanism that decides about the best parent selection for any given node. Other RPL mechanisms, like loop checking and avoidance, local and global repair, etc., should be maintained in order to ensure compatibility with IoT standards.

**Figure 4:**
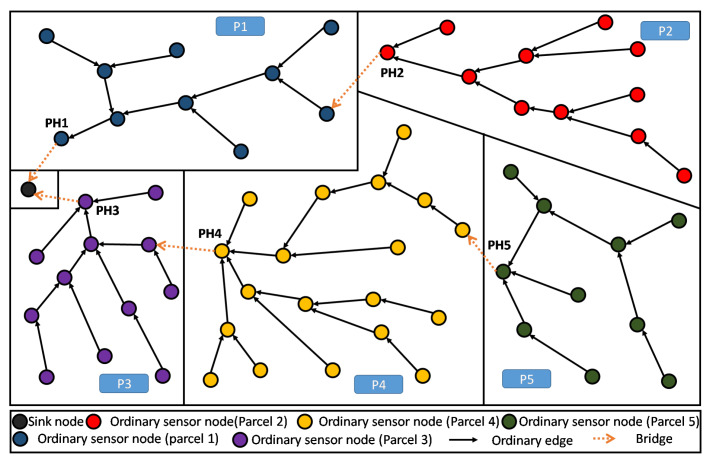
DODAG resulting from Partition Aware (PA)-RPL deployed in a sample farmland—5 duly covered parcels.

As such, our main purpose here is to define an objective function (OF) that enables to build a Partition Aware-DODAG reflecting the physical distribution of crops into parcels in our Farmland. In Figure 4, we show an example of a built DODAG respecting such a property. Each parcel can be seen as a separate land covered by a dedicated sub-DODAG, i.e., the nodes of a parcel belong to a unique sub-DODAG: A parcel respecting this property is said to be duly covered [14]. The complete set of sub-DODAGs compose the overall farmland Partition Aware-DODAG.

By definition in [14], every node in a parcel P that is duly covered will be attached to the same branch or sub-DODAG of the already built DODAG. This sub-DODAG, by itself, forms a destination oriented directed acyclic graph, rooted at a particular and single node $i^{*}$. It is straightforward that all transmitted information from the parcel nodes to the sink transits through this particular node $i^{*}$. In the following, we refer to this particular node (sub-DODAG root) as the Parcel Head (or Partition Head: PH). Moreover, we refer to a Bridge and edge *e* that leaves (considering the UP direction: from leafs towards the DODAG root) a given parcel *P* of the land. Straightforwardly, a farmland parcel *P* is duly covered, if and only if, it contains exactly one single and unique bridge. Both definitions about Parcel Head and bridges are graphically illustrated in Figure 4.

In order to derive this particular structure of DODAG, our proposed algorithm assumes that every node knows the the parcel to which it belongs, i.e., its color, either by knowing its location to derive the color, by hard programming it into the nodes, or by imagining a running initialization phase, where this information is flooded from the sink node towards all the nodes of the network. The proposed improved RPL is based on a novel OF which compares (one by one) the capabilities of the current best parent to all retained parents (i.e., neighbor nodes with higher rank). This function should define a set of tests in order to reach the target objective. For the conventional/standard RPL, the best parent selection decision relies on several common routing metrics usually used for LLNs [24].

These metrics include the latency, the number of hops, and more significantly the Expected Transmission Count (ETX) [24]. This latter is being used in various implementations of RPL [7], where the selection of parents in the DODAG, is made according to the ETX metric, advertised/dispatched through the DIO messages.

The parent selection process, in the standard RPL [5], is triggered every time a potential next hop information is updated. This possibly occurs either upon the reception of a DIO message, or after a timer elapses, or also when all stored DODAG parents are no longer available. The next subsection is devoted to present our parent selection algorithm directing the behavior of our OF.

### 4.2. Parent Selection Algorithm

**Figure 5:**
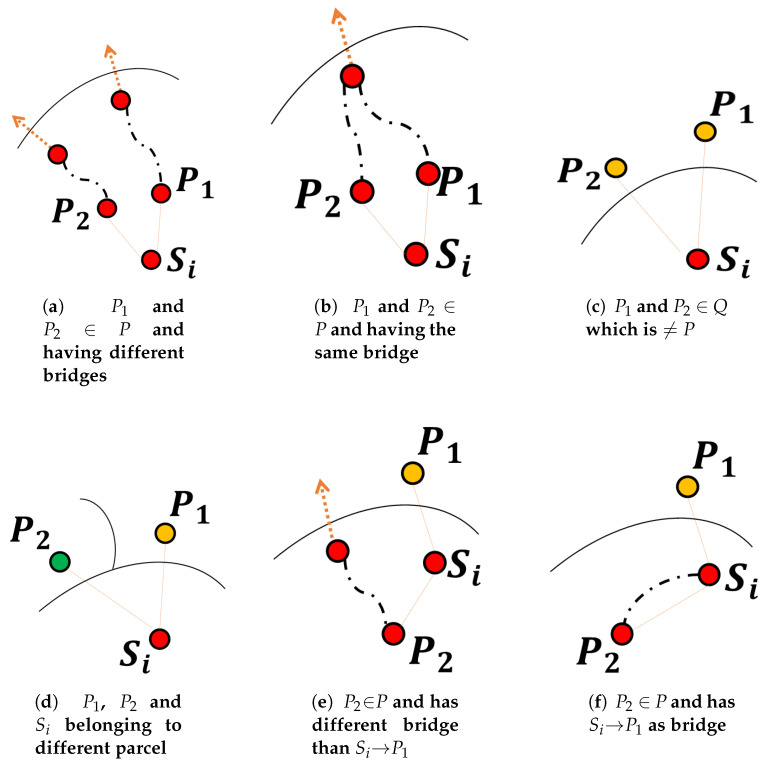
Examples of parent selection situations.

Assume a sensing node $S_{i}$ that belongs to a given parcel *P*. In principle, the parent selection algorithm may face several cases of parents’ candidates. These cases can be categorized in two main situations. The first corresponds to the selection between two potential parents $P_{1}$ and $P_{2}$, located in the same parcel *Q*. The second situation happens when $P_{1}$ and $P_{2}$ belong to two different parcels, namely *Q* and *R*, respectively:

- $P_{1}$ and $P_{2}$ are both in parcel *Q*: in this situation, we can distinguish between three cases:
  (a) *P* and *Q* denote the same parcel and ($P_{1}$, $P_{2}$) using different bridge: In this case, and to foster the creation of one bridge per parcel, $S_{i}$ should select the one going through the best bridge (by comparing the respective ETX values of the bridges as depicted in Figure 5a).
  (b) *P* and *Q* denote the same parcel and ($P_{1}$, $P_{2}$) using the same bridge: In this case, shown in Figure 5b, the decision could be made as usual, i.e., by comparing the ETX of $P_{1}$ and $P_{2}$
  (c) *P* and *Q* are different: In this case, illustrated in Figure 5c, $S_{i}$→$P_{1}$ and $S_{i}$→$P_{2}$ are then both bridges towards the same neighboring parcel. Here again, a usual decision based on the path ETX of $P_{1}$ and $P_{2}$ should be fine.
- $P_{2}$ is in *Q*, but $P_{1}$ belongs to a different parcel *R*: Once again, we can enumerate three different scenarios. The parcel P where sensor $S_{i}$ is located can either be parcel *Q*: (or interchangeably parcel *R*), or different from *Q*: and *R*:
  (d) *P*, *Q*, and *R* are different: Here, $S_{i}$, $P_{1}$, and $P_{2}$ are located in three different parcels. In this case illustrated by Figure 5d, $S_{i}$→$P_{1}$ and $S_{i}$→$P_{2}$ are bridges linking parcel P to neighboring parcels. We may here select the sensor with the best path ETX.
  (e) *P* and *Q* (respectively *R*) denote the same parcel and $S_{i}$ does not belong to the bridge used by $P_{2}$ (resp. $P_{1}$): In this case, $S_{i}$ and $P_{2}$ (respectively $P_{1}$) are in the same parcel, while $P_{1}$ (resp. $P_{2}$) is in a neighboring parcel. As shown in Figure 5e, $S_{i}$ selects $P_{2}$ (resp. $P_{1}$), that is the node located in its same parcel. Again, this is to foster the creation of one bridge per parcel and thus creating a duly covered parcels.
  (f) *P* and *Q* (respectively *R*) denote the same parcel and $S_{i}$ belong to the bridge used by $P_{2}$ (resp. $P_{1}$): in this case, $S_{i}$ and $P_{2}$ (respectively $P_{1}$) are in the same parcel, while $P_{1}$ (resp. $P_{2}$) is in a neighboring parcel. As shown in Figure 5f, if $S_{i}$→$P_{1}$ (resp. $S_{i}$→$P_{2}$) also represents the bridge selected by $P_{2}$ (resp. $P_{1}$), then $S_{i}$ is already selecting $P_{1}$ (resp. $P_{2}$) as the preferred parent and it should keep doing it.

The parcel is identified by a specific color. A node belonging to a given parcel, inherits its color. As previously assumed, this information is accessible by the node itself, say through a method (or function) entitled myColor(). As explained above, the sink node has a different color from the rest of the network. When a node $S_{i}$ has to select the best parent from two node candidates $P_{1}$ and $P_{2}$, the decision is made after performing a set of successive tests as previously described. For this purpose, the node $S_{i}$ requires to know a list of information including: its own color (a), the path ETX value (b), the Id (c), the color (d), the bridge Id (e) corresponding to each candidate.

While local information (i.e., (a)) is easily extracted from the node itself, information regarding all candidates (i.e., (b)–(e)) need to be obtained from the candidates themselves. This can be practically done through disseminating this information into the DIO messages. We can also exploit the Metric container option [5] to dispatch the needed information. In addition, every sensing node has to save the attribute values of its currently used bridge as well. This should be done every time a new preferred parent is selected.

Under the previous assumptions, our proposal can be formulated using Algorithm 1. This Algorithm is executed by every node $S_{i}$ when it has to select one of two parent node candidates $P_{1}$ and $P_{2}$. The meaning of the different variables and methods used in Algorithm 1 are as follows:

- $P_{i}$: denotes a parent node structure which is in our case the DIO message sent by the node $P_{i}$. Note here, that we conflate the parent and its derived DIO message (i.e., both denoted by $P_{i}$).
- $P_{i}.getColor\left(\right)$: a getter to access the color of node $P_{i}$.
- $P_{i}.getBrETX\left(\right)$: a getter to access the path ETX value of the bridge of node $P_{i}$.
- myColor(): a local method, invoked by the sensor node executing the algorithm (i.e., $S_{i}$) to get its color.
- $P_{i}.getBrId\left(\right)$: a getter to get the Id of the bridge of node $P_{i}$.
- $computeETX(P_{i})$: a local method, invoked by the sensor node executing the algorithm (i.e., $S_{i}$) to compute the path ETX if it selects $P_{i}$ as a parent.
- $edgeId(S_{i},P_{j})$: a local method, invoked by the sensor node executing the algorithm (i.e., $S_{i}$) to compute an Id of the edge $S_{i}\to P_{j}$. In our case, this method could be a simple concatenating operation of the two end-points Ids.

In this algorithm, we can locate the two discussed main scenarios: The first goes from line 2 to line 17, and corresponds to two potential parents $P_{1}$ and $P_{2}$ located in a same parcel. The second situation matches the scenario where $P_{1}$ and $P_{2}$ are from two different parcels; and is treated in our algorithm from line 17 to line 39.
**Algorithm 1** BestParent($P_{1},P_{2}$)*This is an OF that selects the best parent between $P_{1}$ and $P_{2}$***if**$P_{1}.getColor\left(\right)==P_{2}.getColor\left(\right)$**then** // $P_{1}$ and $P_{2}$ are in the same parcel $C\leftarrow P_{1}.getColor\left(\right)$
 **if** (myColor()==C) **and** ($P_{1}.getBrId\left(\right)\neq P_{2}.getBrId\left(\right)$) **then** // 1st case: all in the same parcel but $P_{1}$ and $P_{2}$ have different bridges  **if** $P_{1}.getBrETX\left(\right)\leq P_{2}.getBrETX\left(\right)$ **then** // decision based on the ETX values of the bridges   **return** $P_{1}$
  **else**   **return** $P_{2}$
  **end if** **else** // 2nd case: both correspond to bridges or are using the same bridge  **if** $computeETX\left(P_{1}\right)\leq computeETX\left(P_{2}\right)$ **then** // decision based on the best local ETX   **return** $P_{1}$
  **else**   **return** $P_{2}$
  **end if** **end if****else** // $P_{1}$ and $P_{2}$ are located in different parcels **if** $P_{1}.getColor\left(\right)==myColor\left(\right)$ **then** // $S_{i}$ and $P_{1}$ are in the same parcel, and $P_{2}$ forms a bridge  **if** $P_{1}.getBrId\left(\right)==edgeId\left(this,P_{2}\right)$ **then** // ($S_{i}\to P_{2}$) is the bridge used by $P_{1}$   **remove** $P_{1}$ from the neighbor list   **return** $P_{2}$
  **else** // $S_{i}$ switches to $P_{1}$   **return** $P_{1}$
  **end if** **else if** $P_{2}.getColor\left(\right)==myColor\left(\right)$ **then** // $S_{i}$ and $P_{2}$ are in the same parcel, and $P_{1}$ forms a bridge  **if** $P_{2}.getBrId\left(\right)==edgeId\left(this,P_{1}\right)$ **then** // ($S_{i}\to P_{1}$) is the bridge used by $P_{2}$   **remove** $P_{2}$ from the neighbor list   **return** $P_{1}$
  **else** // $S_{i}$ switches to $P_{2}$   **return** $P_{2}$
  **end if** **else** // $P_{1}$ and $P_{2}$ correspond to bridges from different parcels  **if** $computeETX\left(P_{1}\right)\leq computeETX\left(P_{2}\right)$ **then** // decision based on the best local ETX   **return** $P_{1}$
  **else**   **return** $P_{2}$
  **end if** **end if****end if**

### 4.3. Illustrative Example

To better see how PA-RPL runs and builds its DODAG in a way that reflects the farmland layout, we decide to simulate various farmland scenarios. For that, we built our simulation on Cooja [26], known to have multilevel simulation capabilities. We randomly place 150 fixed sensor nodes, emulating the Tmote sky sensors [27], in an arbitrary chosen rectangular farmland of dimensions 400 m × 200 m (i.e., 8 hectares). A sink node, destined to be the root of the DODAG, is placed in position (10,110). We fixed the transmission (respectively interference) range to 50 m (respectively 100 m). Our simulation alternatively runs the standard RPL [5] and the proposed PA-RPL [14], over all the nodes, for sake of performance assessment and comparative analysis.

**Figure 6:**
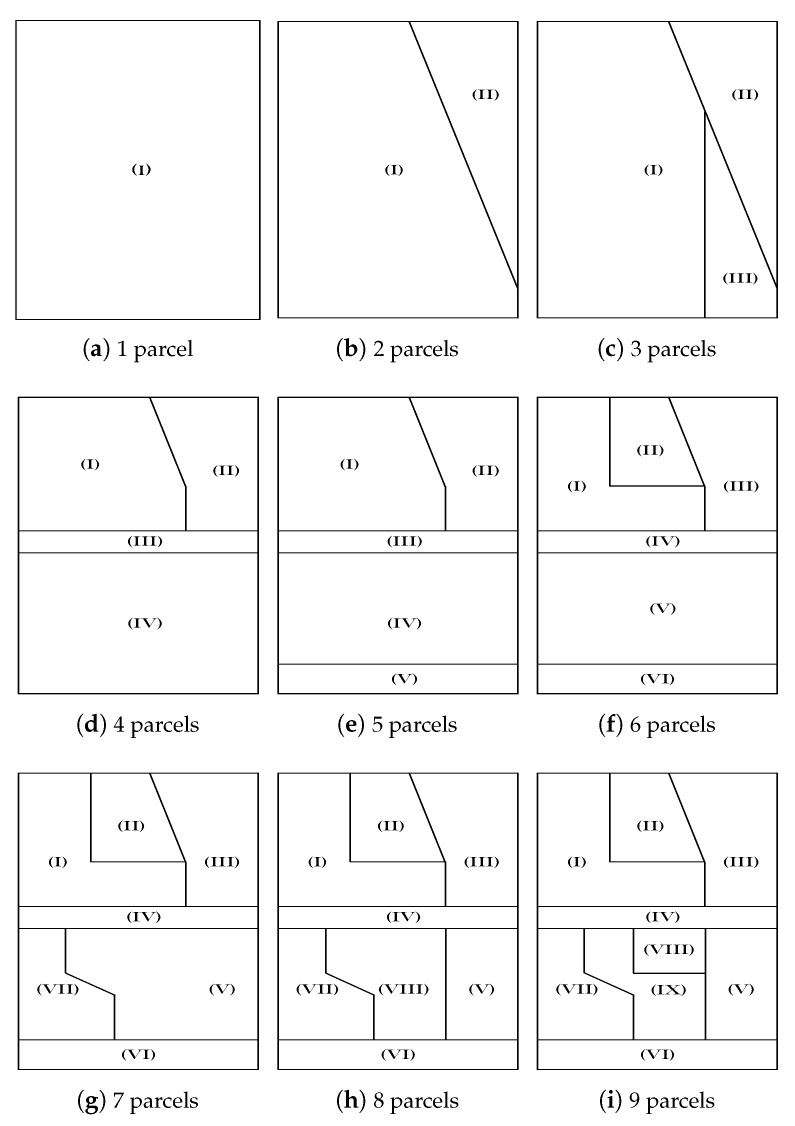

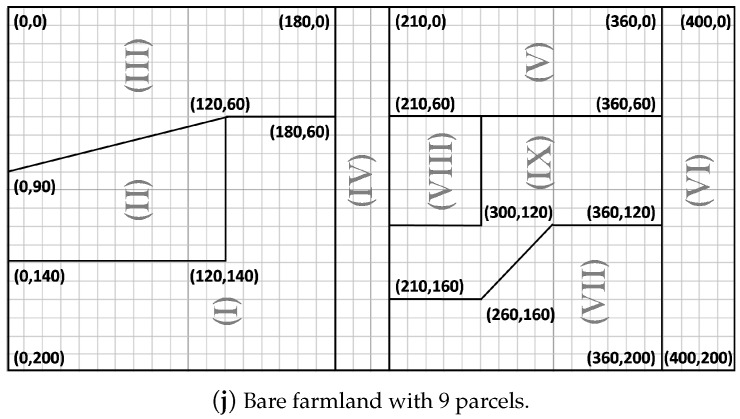
Splitting of the farmland.

The farmland is split into a number of parcels, *N*, that varies from 1 to 9. For each splitting number, the shape of the individual parcels is also taken quite arbitrary (or irregular), as illustrated in Figure 6a–i, corresponding to *N* = 1 to 9, respectively. In Figure 6j we show the whole farmland with nine parcels and the spatial coordinates of their borders.

**Figure 7:**
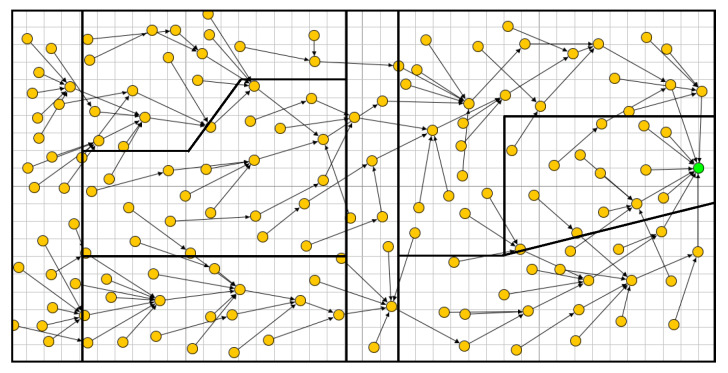
The DODAG built by RPL with 150 nodes.

In Figure 7, we illustrate the DODAG constructed by the standard RPL that is not aware of any limitations between parcels, here 8 parcels are shown. This can be seen as constraint-less DODAG (or blind DODAG), in which the spatial partitioning of the farmland is ignored. In fact, we observe that the communication links, cross the borders between the parcels almost randomly (in any point and any direction), enabling free/unconstrained communication between the nodes that are close to the boundaries. It is straightforward to see that standard RPL does not help the establishment of local (parcel level) processing. This is mainly because the information of nodes, belonging to same parcel, are not necessarily gathered and transferred through the same DODAG branch or sub-DODAG.

**Figure 8:**
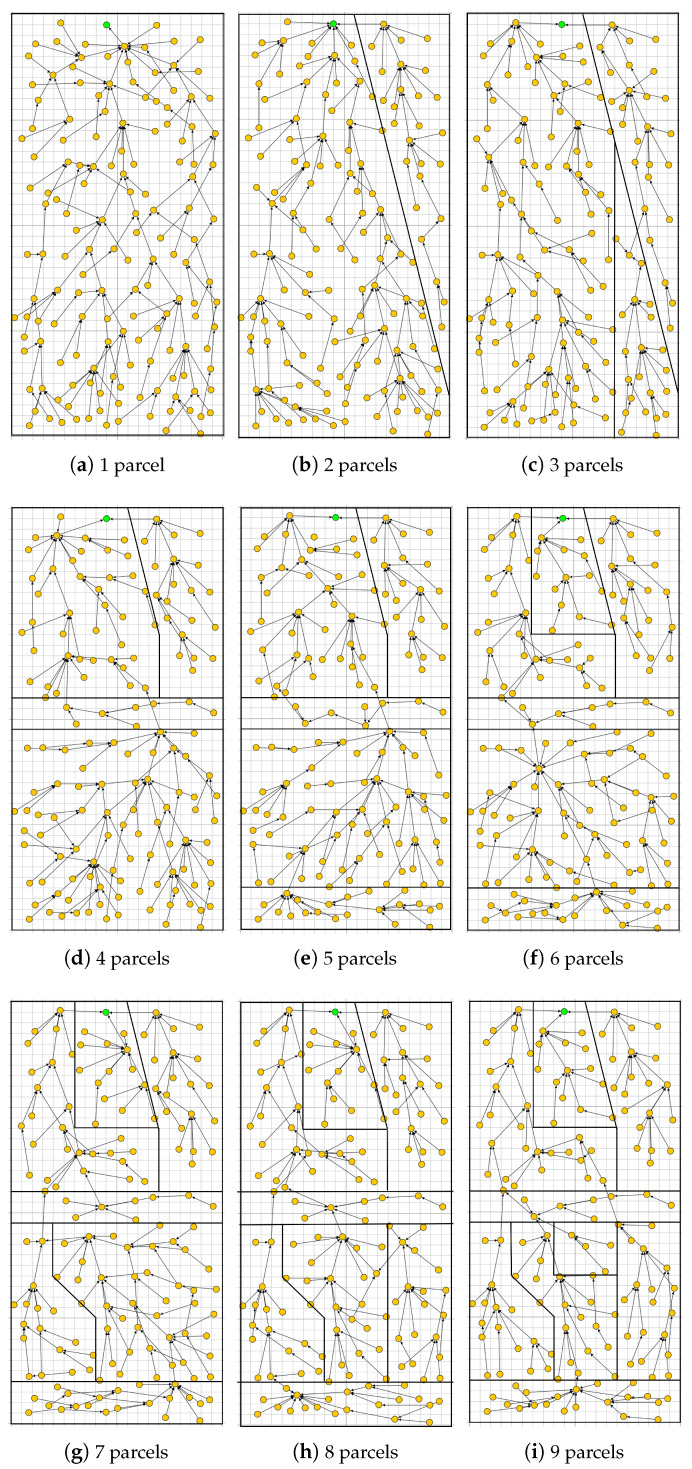
The DODAG built by PA-RPL.

We now apply the PA-RPL assuming the exact same system settings in terms of number of nodes, their type, and spatial location, their transmission and interference ranges, in addition to farmland/parcels’ shapes and areas (i.e., Figure 6). The results are illustrated in Figure 8, where we draw the obtained DODAG for each splitting number N=1,…,9. We clearly see that PA-RPL adapts to the simulated parcel constraints (or the boundaries between parcels), and changes the DODAG structure accordingly. Figure 8a corresponds to the scenario where the whole farmland is considered as a single parcel.

## 5. In-Network Aggregation

When the surface, diversity, and complexity of the farm increase, the number of sensing nodes consequently increases. However, as the number of nodes in the LLN increases, the volume of data substantially increases as well. Therefore, and if we are not careful, our LLN would suffer from different problems such network overhead, traffic congestion, bandwidth consumption, transmission delay, etc.

To overcome many of the aforementioned problems, techniques, such in-network data aggregation, become naturally very attractive, mainly because of their capacity to reduce the amount of induced flowing data inside the network [13]. In fact, in-network aggregation deals with a distributed processing of data at the intermediate nodes within the network, mainly executing aggregation functions. There are mainly two approaches of in-network aggregation, namely lossy and lossless:

- lossy in-network aggregation is the approach that combines and processes the data received by a sensor node from its neighbors by performing a lossy aggregation function such as MAXIMUM, AVERAGE, MINIMUM, etc. In this case, only the output of this function is forwarded up instead of the whole information.
- lossless in-network aggregation refers to the process of merging or compressing the data received from different source nodes into a single data packet, i.e., the whole information is concatenated and then forwarded up.

With the help of these approaches we can decrease the number of data exchanges in the LLN. Therefore, it increases the life time of the network and reduces its energy consumption. In order to aggregate data flowing from the source nodes towards the sink, the first step is to elect some special nodes that operate as aggregation points (or aggregators) and define a preferred path for forwarding data. Straightforwardly, data aggregation techniques are tightly coupled with how aggregator nodes are elected, how the data is gathered, as well as how aggregated packets are routed through the network to the sink. Then, one of the key elements of in-network aggregation deals with the problem of building a routing topology that facilitates aggregation.

### 5.1. Standard RPL and Its Limitations

**Figure 9:**
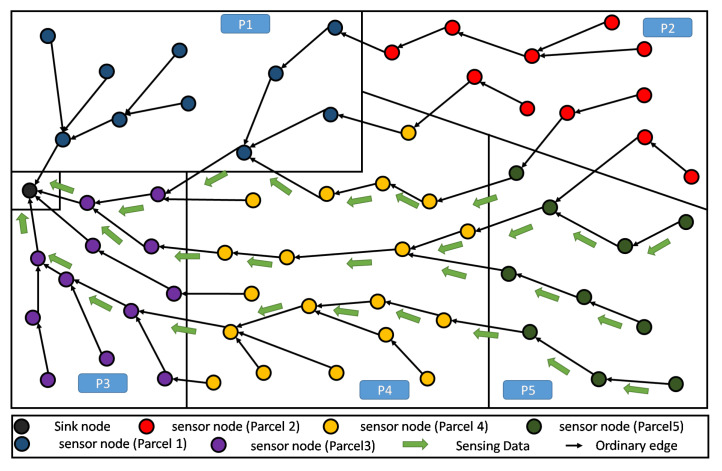
Standard RPL DODAG: difficult to support aggregation at parcel level.

**Figure 10:**
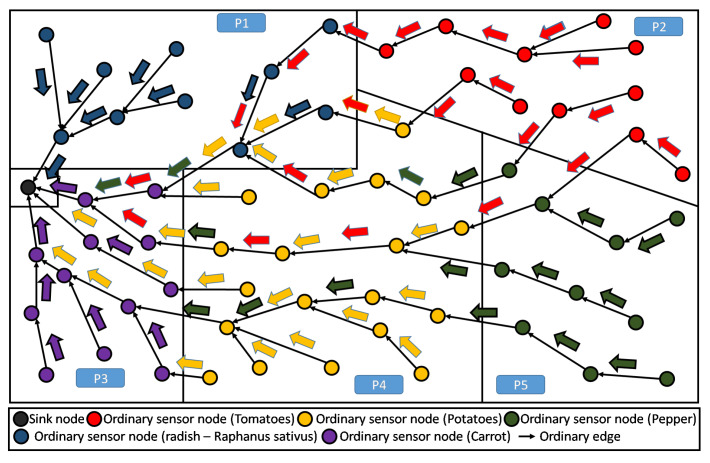
Standard RPL DODAG: high data heterogeneity decreasing the aggregation efficiency.

When using RPL, the communication is driven by the constructed DODAG, see Figure 3. Recall that this DODAG is incrementally created/built based on an objective function (OF) to be satisfied. In principle, the standard RPL intrinsically enables a certain level of aggregation. However, in the context of farms that are composed of several parcels, the aggregation feature is no longer beneficiary nor practical, see both cases below:

- Need of lossy aggregation at parcel level: It is worthy to note, that most smart-farming decision support system (DSS), need parcel information that results from lossy type aggregation. As an instructive example, in greenhouses, DSS exploits periodically collected information about climatic parameters, in order to find the most suitable growth conditions for the cultivated crops. In the case of strawberry, we need to maintain the temperature at 30 ${}^{{}^{\circ}}$C during the day and 15 ${}^{{}^{\circ}}$C during the night, for one year. Straightforwardly in this context, only the minimum and maximum temperatures of the sensed data are required by the DSS in order to control the greenhouse temperature. As shown in Figure 9, standard RPL makes the generated data flowing from all nodes, converging to the sink, without any consideration of the parcels’ boundaries. This prevents from any local aggregation function to be applied at the parcel level.
- Lossless aggregation for highly heterogeneous LLN: When the farmland is cultivated by different crops, the proposed topology provides efficiency for performing lossless data aggregation, (such as fusion and compression). This is due to the high heterogeneity of the collected data. Obviously, in standard RPL the flow of data messages from different parcels, though different crops, will be of high heterogeneity because of the several kinds of collected parameters, their frequencies of measurement, etc. Inherently, the application of any compressing/fusion algorithm on this heterogeneous data will give a poor efficiency. The Figure 10 gives an illustrative example of this situation.

This motivates the need for a built DODAG that reflects the physical distribution of crops over the parcels: it turns out that in-network aggregation is easier to implement for smart farming applications whenever this property is achieved. Luckily, our proposal, PA-RPL builds its DODAG to perfectly suit this spatial distribution, which makes it a perfect fit to support in-network aggregation. In the literature, and to the best of our knowledge, none of the proposed OFs paid enough attention to these partitions, but PA-RPL.

### 5.2. PA-RPL and In-Network Aggregation

As a result of its capacity to reflect the spatial distribution of crops over the parcels, PA-RPL solves most of the needed ingredients to easily implement in-network aggregation: electing special nodes as aggregators, and defining preferred paths to route data towards these aggregators or to the sink. In fact, gathered data of a target parcel will converge to the Parcel Head node (by construction), and thus, in-network processing operations can be easily executed in each sub-DODAGD’s root, i.e., PH node.

**Figure 11:**
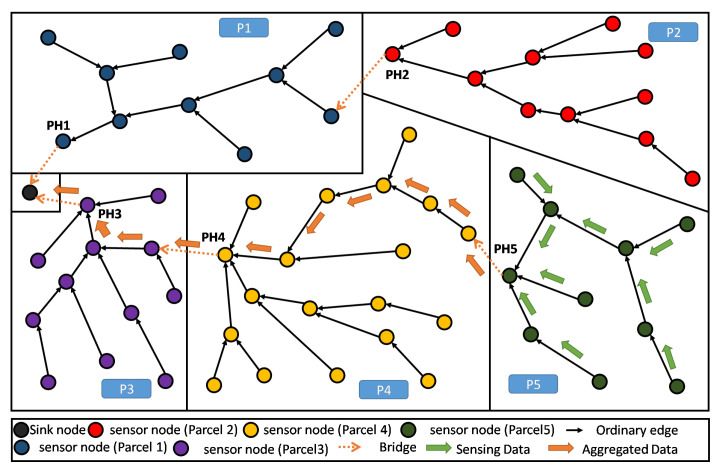
PA-RPL DODAG: collection and aggregation of homogeneous data at parcel 5.

As shown in Figure 11, periodical gathering of the entire parcel data in one elected aggregator node (i.e., the Parcel Head), can simply be processed using a lossy aggregation function like maximum, minimum, average, etc., then only the result will be sent to the sink.

Moreover, when the farmland is hosting different crops distributed over different parcels, the proposed topology enables an efficient lossless data aggregation function like fusion and compression. This contrasts with standard RPL in terms of efficiency because of the high homogeneity of data within the parcel. Obviously, in one parcel it is common to have one kind of crop, presenting the same kind of parameters to continuously monitored, and requiring quite similar kind of decisions to be made. As shown in Figure 11, homogeneous data collected in a single parcel (P5) will be all gathered and processed at the Parcel Head (PH) then only the output message is forwarded up to the sink.

## 6. Case Study: Potato Pest Prevention

In the literature, applications using wireless smart sensor/actuator networks (WSN) in potato farms are quite widely proposed and applied. In fact, starting in 2005, the Lofar Agro [28] project aimed to apply sensor networks in protecting the potatoes plantation from a fungal disease known as the $\ddot{P}$hytophthora Infestants. In [29], an experimental WSN system for improving the cultivation of the potato crop is described. The proposed system makes important saving in terms of resources used in cultivation including fertilizers and irrigation water. In [30], PotatoSense, a precision agriculture application for monitoring a potato plantation field in Mauritius, is described. Different energy efficient algorithms are used in the proposed system to ensure that the system lifespan is prolonged. Recently, PotatoNet [31], that is an outdoor testbed for IP-enabled WSNs (LLNs), has been developed. It is designed to operate without on-site maintenance for extended periods of time.

In this section, we are interested in the Potato Late Blihgt Pest. Thus, we consider realistic scenarios of a Potato Late Blight Pest Monitoring Application, where PA-RPL can be used to gather relevant data that help preventing favorable conditions for this disease to start. The late blight is considered to be the most devastating potato epidemic pests, and caused by *Phytophthora infestans*. Historically, this epidemic is known to be the main cause of the Irish famine in the 1840s [32]. In addition, and according to a recent study, 21% of the worldwide loss in cultivation of potatoes is attributed to the devastating late blight disease [33]. In this section, we start with describing such an application, and then, we move to evaluate the performance of our protocol in comparison with the standard RPL.

### 6.1. Potato Late Blight Pest Prevention Application

The late blight is one of the most feared potato epidemic pests by farmers, as it can completely destroy the potato crop within few days if the weather is conducive for disease progress.

**Figure 12:**
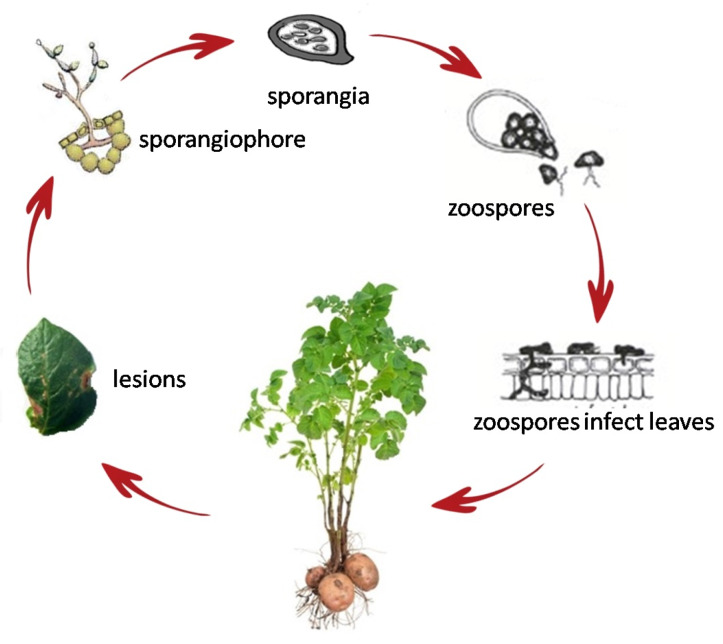
Phytophtora life cycle.

It is caused by *Phytophthora infestans* fungus-like oomycete. The *Phytophthora Infestans*, presented by Figure 12, has a complex structure similar to a microscopic plant. Its leaves, stem, and roots form its mycelium, through which, the pathogen reproduce. The mycelium grows on the stems, tubers, and leaves of the host plant, mainly a potato plant, or even on its residues. The fungus fruit, called sporangia (located in the sporangiophore as shown in Figure 12), can be carried by wind for up to 60 km of potato growing regions [33], leading to rapid spread on wide-scale of this pathogen. In addition, these sporangia contain each 8 zoospores, a sort of swimming seeds, allowing a rapid spread of the disease by rainwater. To better see how fast such a pathogen can spread, it is estimated that from a single lesion, may be produce up 300,000 new sporangia daily.

Usually, the pathogen is dormant, waiting for the right weather conditions to wake up. In fact, only during specific weather conditions, a temperature around 13–15 ${}^{{}^{\circ}}$C and a relative humidity (R.H.) of 90–100%, that sporangia release their zoospores. Thus, controlling these conditions leads to controlling the spread of the disease. In modern potato farms, late blight disease is commonly treated using a set of chemical fungicides. To optimize the number and doses of fungicide applications per season, numerous decision support systems, based on late blight forecast models, have been developed [34]. As expected, these models are based on climatic data, mainly temperature and humidity. The goal is either to react proactively by avoiding to meet the late blight adapted weather conditions (i.e., in case of greenhouses), or re-actively, by the application of fungicides quickly before the disease spreads out (i.e., in case of open potato fields).

The use of weather data for late blight forecasting has long been a common practice by farmers. In 1951, Wallin [35] set in place a first empirical forecast model based on weather data used for blight gardens. This model assumes that the disease evolution is proportional to the duration of exposure of the plant to a humidity higher than 90% in certain interval of temperature. Later, the Wallin’s model was some improved giving what is called the *SIMCAST* forecast model [36]. The model describes the degree of development of the Phytophthora Infestans in potato by computing an equivalent risk factor called Blight units. This risk factor is computed based on the number of successive hours where relative humidity is >=90%, and the average temperature belonging to any of these respective ranges: (<3${}^{{}^{\circ}}$, 3–7${}^{{}^{\circ}}$, 8–12${}^{{}^{\circ}}$, 13–22${}^{{}^{\circ}}$, 23–27${}^{{}^{\circ}}$, and >27${}^{{}^{\circ}}$).

Based on the SIMCAST forecast model [36], many decision support systems were developed to prevent from potato late blight, such as euroblight [37], blightPro [34], and castor [38]. In these systems, the cumulative estimated blight units are used in order to make decisions about the timing/scheduling of fungicide application on the plants.

Recall here, that the goal of this section is not to create a more effective prediction model, or a better schedule for fungicide application. We are rather interested in providing an effective, long life, and reliable sensor/actuator network that can effectively support such existing support decision systems, or more precisely, an effective management of such network.

### 6.2. Performance Evaluation without In-Network Aggregation: PA-RPL vs. RPL

After presenting the late blight pathogen, we move to describing our considered scenario where a wireless sensor/actuator network is deployed. We thus assume that we have a farmland similar to the one described in Figure 6j, where we grow potato crops of different types or planted at different times, along with other kinds of crops. A set of nodes (sensors/actuators) are uniformly distributed across the farm. At this stage, our goal is to evaluate the performance of PA-RPL in comparison to the standard RPL, when a standard centralized data collection application (i.e., a conventional support decision system) is running at the sink node, i.e., without any in-network processing. It is clear that this is not the scenario where PA-RPL performs best. In fact, the load balancing of the DODAG is potentially affected by the spatial partitioning of the farmland and the nodes distribution. However, through this study, we try to answer two main questions corresponding to two main case-studies:

- In comparison with RPL, how effective is PA-RPL in a scenario where all parcels of the farmland are monitored simultaneously?
- In comparison with RPL, how effective is PA-RPL in a scenario where only few parcels of the farmland are monitored simultaneously?

#### 6.2.1. Case 1: All Parcels Are Simultaneously Monitored

For this scenario, we assume that all monitored parcels are growing potatoes, and that the number of nodes might cover all the parcels in the farmland. Our goal here is to push the PA-RPL to its limits and see how it would perform if we need to collect data, at the same sustained rate and from all parcels in the farm (i.e., draw a theoretical lower bound on the PA-RPL performance). To evaluate the performance of PA-RPL in comparison with RPL, we use simulation. For that, we use the simulation parameters of Section 4.3. Moreover, we assume that the nodes transmit their sensed data periodically every 30 s. The evaluation of the PA-RPL consists of exploring the evolution of some metrics as a function of time. In the simulation, these metrics are evaluated for a variable number of parcels in the farmland. Four key performance metrics are considered in this evaluation:

- Convergence time: This is the time required for the algorithm to build the DODAG and reach a stable state, i.e., after that time, the DODAG remains almost unchangeable. This convergence time can be measured by monitoring the number of preferred parents switching along then simulation. When the permanent regime is reached, this number becomes almost constant.
- Communication overhead: this parameter represents the communication load used for the DODAG maintenance messages (i.e., control packets used to establish and maintain the DODAG). In LLN, a good performance routing algorithm must generate the lowest number of maintenance messages in order to leave most of the communication bandwidth for the data packets.
- Packet delivery ratio (PDR): this parameter helps to evaluate the reliability of the LLN. In fact, the PDR represents the percentage of the received messages out of the transmitted messages. This parameter reflects the reliability of the protocol and determines the usefulness of any network.
- Global energy consumption: through this parameter we study the evolution of the energy consumption of the network as a function of time, and the relation between this energy and the number of parcels. In the LLN, it is well known that most of the energy consumption is related to communication. Hence, instead of energy, it is commonly accepted to exploit the percentage of time, in which communication transceiver is active.

**Figure 13:**
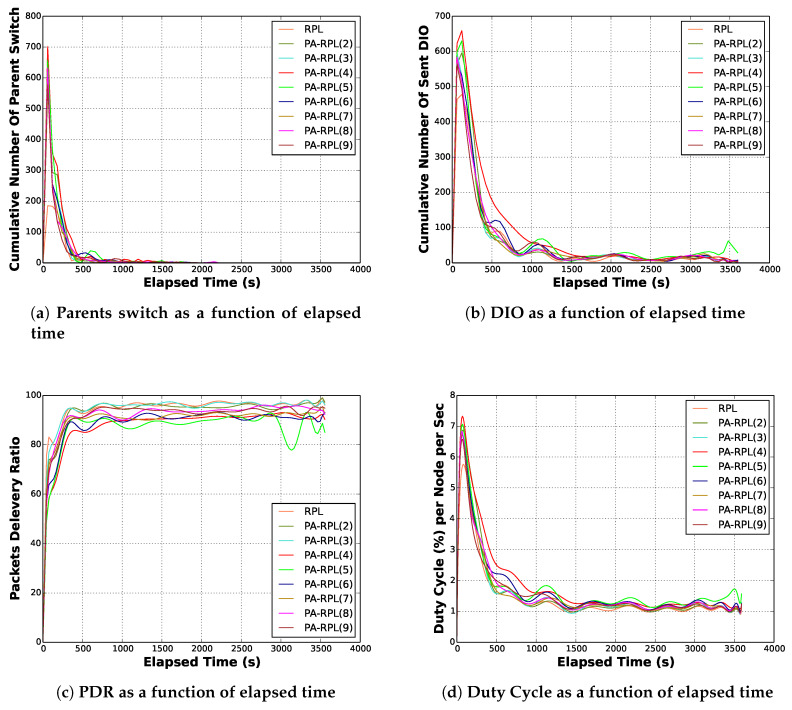
PA-RPL performance as a function of elapsed time.

The simulation results in terms of the four defined metrics are presented in Figure 8. The Figure 13a shows that the number of best parent switching starts very high in the first simulation minutes, then rapidly decreases to finally reach a negligible value after 1500 s to be considered as the convergence time. Practically, after that point of time the DODAG becomes almost stable and conserves essentially the same shape. From the figure, it is clear that the convergence time does not depend on the number of considered parcels. In addition, we can see that both protocols have similar permanent regime. However, and even if both transient regimes last for the same duration, we can see that PA-RPL induces more parent switching before converging in comparison with the RPL. This can be explained by the additional constraint in our best parent selection process, in order to have one bridge from every parcel. In a smart farm, where an LLN is used to operate for months and years, the convergence duration of 1500 s is a very acceptable value for both protocols, as once permanent regime is reached, almost all the available bandwidth (and energy) will be used to transfer data.

In fact, Figure 13b, confirms that most of the exchanged DIO messages (i.e., the overhead) are generated before the network convergence (during the transient time) then they become negligible. In addition, we can see that during the transient time, the number of DIO messages generated by the PA-RPL is higher than the one generated by RPL, before both overheads decay towards zero in the permanent regime. Another important remark is that the number of DIO messages is not directly related to the number of parcels.

As expected, the energy curves, depicted in Figure 13d, present similar shapes as those of parent switches and sent DIOs. We can easily identify the transient and permanent regimes. In addition, Figure 13d illustrating the duty cycle per node per second as a function of the elapsed time, shows that both protocols consume almost the same amount of energy and that this energy consumption is almost independent of the number of parcels in the farmland (i.e., 1 to 9).

Finally, Figure 13c depicts the evolution of the ratio of received packets over time. Similar to the previous metrics, we observe two operation regimes. During the first (transient) regime, the ratio of received messages is very low, however during the permanent regime, this ratio substantially increases around 90%, hence reaching standard RPL performance. This is obviously despite the important intrinsic difference between both resulting DODAGs.

In conclusion, we can say that PA-RPL, behaves similarly to the standard RPL even when pushed to its limits and where we are not really making use of the key advantage of reflecting the spatial partitioning of the farmland. The advantage of PA-RPL over the standard RPL will be presented in the next section.

#### 6.2.2. Case 2: Few Parcels Simultaneously Monitored

For the second case, we consider a more realistic scenario. Given that each type of crop grows during a particular season and observes a number of evolution phases, a good monitoring application shall take such variability into account. In fact, along the evolution phases, crop monitoring requirements vary in terms of type of information to gather, and periodicity of data collection. As such, in the same multi-crop farm, it is common to have different data collection frequencies simultaneously; raised from different parcels. Each frequency reflects the crop evolution phase and season. When the collection frequency of a given parcel is very low, nodes belonging to this parcel will be in a sleep mode for quite long periods (i.e., further saving precious energy). However, when high frequencies of data gathering are applied, nodes will wake up for more time and thus consume more energy. Moreover, even during these “requiring-high-frequency” phases, it is possible to turn nodes into sleep mode for longer periods of time with no risks. For example, after the irrigation of a parcel, it is reasonable that the soil moisture collecting frequency gets extremely reduced.

With such realistic scenarios, the superiority of PA-RPL over RPL becomes more tangible. Indeed, the standard RPL clearly forces the nodes of one culture (or one parcel) to wake up and function during the season of another culture (or another parcel), in order to route the information. This may clearly cause an important loss of energy. In fact, based on the radio duty cycle mechanism [39], a node sleeps most of the time and periodically wakes-up to check if a radio transmission activity is detected. In this case, the node stays awake to listen to the communications. With RPL, a communication message arising from one parcel will take several paths before reaching the sink node. Obviously, all the nodes belonging to the routing trajectory, in addition to their neighbors, will detect this communication activity. Therefore, an important number of nodes remain uselessly awake. In contrast, the PA-RPL will choose a unique trajectory linking the Parcel Head (PH) to the sink, hence avoiding the messages to be dispatched to a large zone of the farm LLN. This saves considerable amounts of energy because large number of non-concerned nodes will not need to listen to non-pertinent communications, i.e., they do not disturb their sleeping.

**Figure 14:**
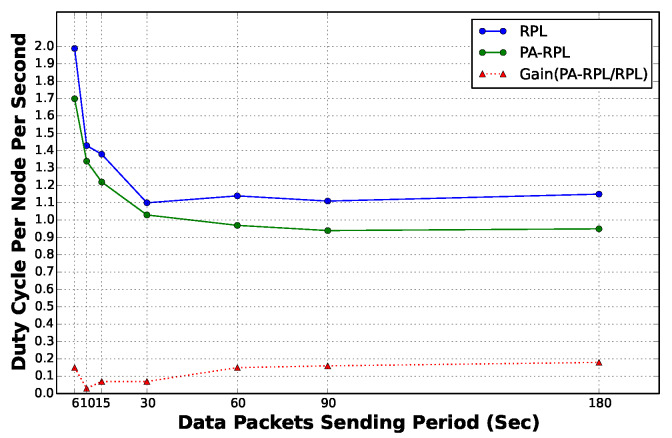
Gain as a function of sending period.

In order to compare the performance of PA-RPL and RPL we consider periods of time, or scenarios, where only some of the parcels have crops that need monitoring. For instance, let us consider the case where only nodes located in parcel VI need to transmit data to the sink node. We then vary the applied data load and measure the duty cycle per node per second in permanent regime (i.e., after the 1500 s transient time). The results are presented in Figure 14. We observe that when the sending frequency is high, the energy consumption goes up for both protocols. As sensors reduce their sending frequency, the energy consumption is also reduced, and stabilizes at an average of 1.12% per node/sec for RPL against 0.92% per node/sec for our PA-RPL; thus performing a gain of almost 20%.

Recall here that deployed sensor nodes are limited in capacity and resources. Their role should be reduced to collecting data and making sure to convey it to the sink node where all the complexity resides. Moving the logic and the control (e.g., seasonality effect) to the application layer of each node makes the nodes more resource demanding. This explains the biggest contribution of PA-RPL that is its ability to reduce the overall energy consumption. Energy savings at the scale of the WSN are achieved by minimizing the global duty cycles. The duty cycle indicates the frequency with which the node wakes up to hear (whenever there is radio activity around it) or send data. With PA-RPL, we manage to minimize the global duty cycle by routing the data flow via paths avoiding unnecessary radio wake-ups of nodes in distant parcels. This resulted in an overall energy saving of around 20%. Implementing the logic at the application level will simply lead to loosing this property (limiting the impact of gathering data and sending it to the sink node), which means that the messages sent by nodes inside a parcel will be disseminated throughout the complete agricultural field, waking up almost all the nodes.

### 6.3. PA-RPL with Parcel Level In-Network Aggregation

We recall here that the main advantage of PA-RPL is to build a specific routing topology which enables an efficient data in-network aggregation. The latter is a well-known technique that helps to reduce the data communication load. This technique can be classified into two types of approaches: lossy and lossless. Lossy in-network aggregation is the approach that combines and processes the data received by a sensor node from its neighbors by performing a lossy aggregation function such as MAXIMUM, AVERAGE, MINIMUM, etc.

It is worthy to note that most agriculture applications do not require all the sensed data, but only local lossy aggregated values instead. Moreover, this technique is generally applied in conjunction with a clustering algorithm that assembles a number of nodes in a cluster and then selects a cluster head, in which the aggregation will be executed. Again, we can clearly see the superiority of PA-RPL over RPL as it insures an easier clustering as well as an easier implementation of lossy in-network aggregation, by simply designing the Parcel Head (PH) as a cluster head, and applying the aggregation operation on data collected from sensors having the same color as the cluster head (i.e., belonging to the same parcel).

#### 6.3.1. Implementation of In-Network Aggregation for PA-RPL

In this section, we describe an easy implementation of a lossy in-network aggregation for PA-RPL to help fight against the Late Blight pest. In our considered case scenario, it is important to note that because of the epidemic nature of the late blight pest, both humidity and temperature are required to be continuously measured in closed locations/points through the parcel area. In fact, whenever the pest conditions (humidity and temperature) are met in a single sensing point, late blight develops and promptly propagates to neighboring areas, and hence quickly covering the whole farm. Likewise, the only required input for a late blight decision support system is the range value of temperature and the maximum value of humidity through the monitored parcel. Then, it is clearly sufficient to apply a local processing algorithm in order to compute/detect the maximum value of humidity (i.e., exceeding a predefined warning threshold) through the whole parcel, and to transmit only this information to the decision system. Similarly, for temperature, the local processing algorithm has to compute and identify the minimum and maximum values then transfer them to the decision algorithm.

In this Algorithm 2, once a node receives a message, it checks its own status, i.e., either a Parcel Head (PH) or a Normal Node (ND). In the former case, the node applies all its local processing routines (i.e., computing and aggregation) whenever the received message is created by a node belonging to the same parcel (i.e., of the same color as the PH). However, in the latter, the node simply forwards the information to its preferred parent. In the case of PA-RPL, the created DODAG ensures that all the data will be gathered in the PH where it will be processed. Only the aggregated/processed information will be then transmitted to the sink, hence highly limiting the exploited bandwidth and energy. Recall that in standard RPL’s DODAG, it is clearly not possible to apply such processing.
**Algorithm 2** RcvMsgCallback(Msg)1:*This is a receive Message Callback in aggregation scenario*2:Payload←getPayload(Msg)3:**if**(isParcelHead())**and**(Msg−>getColor()==myColor())**then** // This is a Parcel Head4: humidity←getHumidity(Payload)
5: temperature←getTemperature(Payload)
6: UpdateMaxHumidity(humidity)
7: UpdateMaxTemperature(temperature)
8: UpdateMinTemperature(temperature)
9:**else**10: PreferredIpAddress←getPreferredIpAddress()
11: SendMsgTo(PreferredIpAddress, Payload)12:**end if**

#### 6.3.2. PA-RPL Performance in Potato Pest Prevention Application

In the present subsection, we evaluate the performance of the proposed PA-RPL in terms of energy consumption in the context of local (parcel level) lossy in-network aggregation. To better assess the gain in efficiency that PA-RPL can bring, we simulate various scenarios where PA-RPL is applied against similar scenarios using the original RPL. For that, we consider the farmland described in Figure 8i, and assume that the potato crop is planted in parcel number VI. The local processing described above, for humidity and temperature, will be executed in the Parcel Head (PH) node.

**Figure 15:**
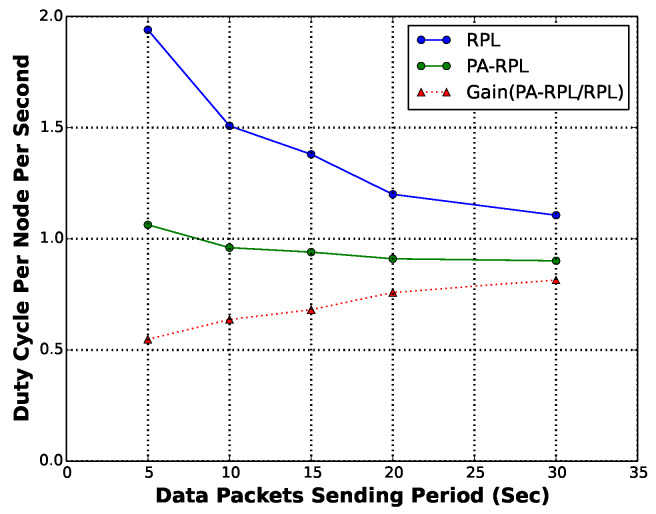
Gain as a function of sending period (in-network aggregation scenario).

In order to efficiently gather this data in PH, we implement and deploy an application level forwarding algorithm in all nodes of the parcel. In this evaluation, we are mainly interested in the gain in terms of energy consumption for different collecting/reporting periods. In Figure 15, we show that PA-RPL consumes less energy in all scenarios, compared to RPL. We also show that the gain in energy consumption rises up to 40% in high frequency scenarios, if aggregation is applied. An other interesting finding is that PA-RPL is less sensitive to the increase of the data sending period (i.e., high frequency monitoring scenarios) compared to RPL. This means a better flexibility and usability to meet different types of applications, configurations and scenarios.

## 7. Discussion

One of the main goals of our proposed routing algorithm, PA-RPL, is to enable a more efficient parcel/partition level in-network aggregation. This is achieved by mapping the created DODAG, to the sub-divisions or parcels of the farmland. In addition to the above mentioned performance results, it is important to know the other set of benefits that would be gained from this partition aware routing algorithm. In this section we expose rooms of optimization enabled by PA-RPL.

**Figure 16:**
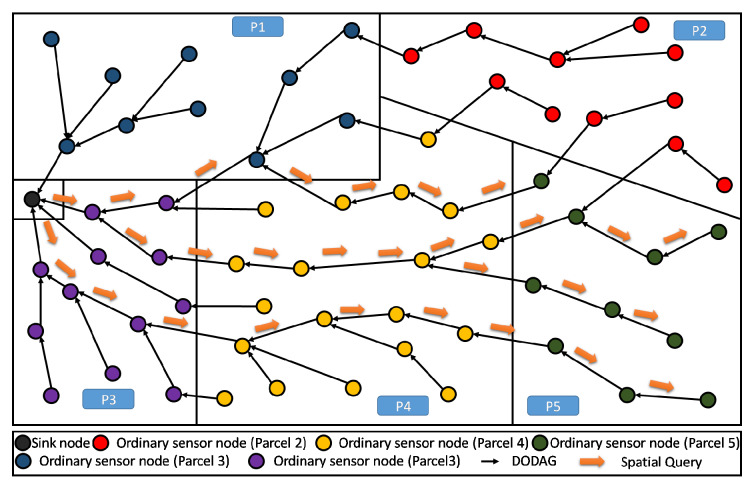
Spatial queries dissemination with RPL.

**Figure 17:**
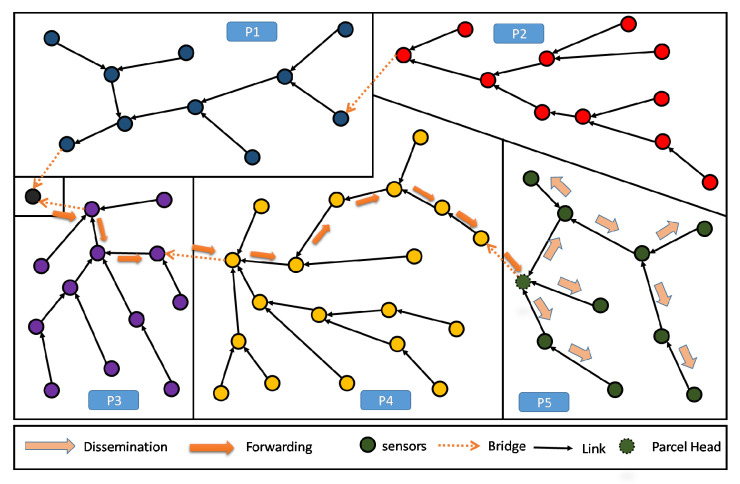
Spatial queries dissemination with PA-RPL.

**Figure 18:**
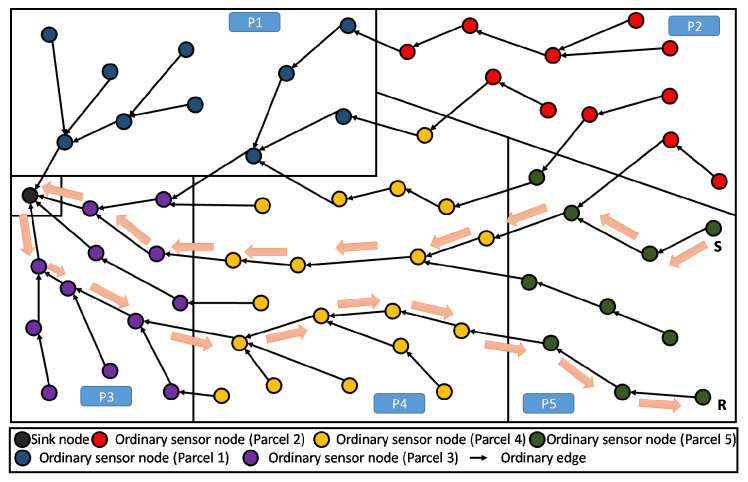
Intra-parcel message exchange with RPL.

**Figure 19:**
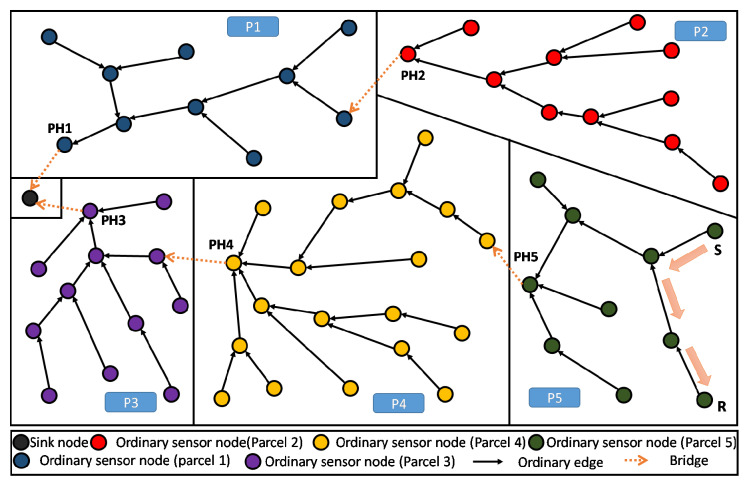
Intra-parcel message exchange with PA-RPL.

Let us begin by exploring an instructive example of the different data streams in the smart farm network. For instance, in the same farm, three types of data-streams may simultaneously exist: (i) MP2P data-streams collected from sensor nodes deployed into greenhouses, stables, parcels, etc. (ii) P2MP data-streams such as control messages from the sink to the field, including: electro-vanes, air conditioners, milking machines, gates, agriculture robots, drones, etc. (iii) P2P communication such as exchange between local (parcel level) decision system and local instruments (air conditioner, local alert system, fertilizing machines with nodes in the same parcels, communication and coordination between two automated tractors, etc.). Based on the latter example, it becomes clear that there are mainly three data stream patterns in the smart farm network:

- Parcel to Sink: this kind of data stream represents the data messages collected from agriculture parcel nodes and forwarded to the sink. As showed above, PA-RPL enables an easy implementation of an efficient in-network parcel data aggregation and processing in the upstream traffic, which highly reduces the effective amount of data that needs to be transferred up to the sink.
- Sink to Parcel: this kind of messages, used to be either control or query messages that come from the sink and disseminate through the network in order to reach parcel/partition nodes. With RPL, this kind of message will proliferate through the farm LLN following different ways before reaching the target section, see Figure 16. Hence, this induces a useless energy consumption. On the other hand, PA-RPL enables a more efficient approach. It consists of selecting the best way between the sink and a target parcel, then transmitting only one message through it. As illustrated in Figure 17, upon reaching the first node in the parcel, the message will be broadcasted to the other nodes within the same section.
- Intra Parcel: this kind of messages in SF are used to have the highest proportion of the (Point-to-point) communication. In fact, in agriculture, it is frequent to find machines, operating in the same parcel that need to communicate to each other and exchange information. A practical example of point-to-point communications may correspond to a drone [40] that receives information directly from the field deployed sensors or a communication between tractors covering the same parcel. This is of capital importance, as handling dynamic scenarios by RPL is becoming a very active topic lately. Many recent publications treat of the same subject [41,42,43], and are all about possible improvements/adaptations of RPL in order to support mobile nodes (e.g., tractor, drone, and robot). Likewise, PA-RPL can be adapted to support these dynamic scenarios. Nevertheless, we can expect this adaptation to be relatively easier compared to RPL. In fact, with PA-RPL, all the parcel nodes are assembled in the same DODAG branch (sub-DODAG) allowing to easily gather information about a parcel at the Parcel Head node, reducing the effort needed to make the mobile node collect this data. Additionally, if we assume One-to-One communication scenarios (where the communication needs to go UP then DOWN) covering situations where a node exchanges data with the mobile node, PA-RPL turns out to be more suited than RPL as illustrated in Figure 18 and Figure 19. Indeed, the mobile node can easily integrate the built DODAG by simply acquiring the color of the parcel. As such, the mobile node simply needs to move from one parcel to the other while adopting each time the color of the visited parcel. Moreover, the One-to-One communication can be easily performed by going Up to the Parcel Head (no need to go up to the sink), then Down to the target node. This makes the whole exchange process run locally (involving the mobile node and nodes in the parcel solely), which makes it possible to support more than one mobile node in the network simultaneously.

From the above, it becomes clear how important to take into account the partition of the farm into the DODAG building process in order to optimize the whole communication system. Indeed, when the built DODAG allows an easy gathering of all the data messages of a parcel, in the same node (Parcel Head), it enables efficient in-network aggregation. Moreover, when a selected (or elected) node to collect data, is a common parent for all the parcel nodes, it allows more efficient intra-parcel (or partition) communications. In addition, when the common parent selection process is based on the path reliability, between this node and the sink, all the sink-to-parcel messages will follow the same way. On the other hand, the fact that farm partitions are usually very autonomous and/or independent motivates the need to local decision making processes, in contrast to all-centralized systems. One of the main attractive features of the proposed PA-RPL [14] is the fact that the built DODAG straightforwardly enables parcel-level information processing.

However, it is mandatory to pay attention to the fact that this PA-RPL DODAG, will potentially drain the energy of the nodes located between the Parcel Head (PH) and the sink. This is usually referred as Hot-Spot problem [44], leading to a decrease in the LLN lifetime. This is an attractive topic of research that requires specific careful investigation and countermeasure solutions. In principle, lifetime improvement solutions, inspired from similar literature situations, are very feasible and applicable to our algorithm. In fact, the objective function of the PA-RPL can be extended, in order to take into account the node residual energy, in the parent selection process. For instance, a potential solution can be based on the frequent change of the Parcel Head and other end nodes, upon reaching a threshold of residual energy. This dynamic change in roles between nodes helps to balance the energy consumption between them, hence increasing the network lifetime.

Although PA-RPL is conceived in order to give an appropriate answer for SF routing requirements, we believe this will be applicable for a number of other IoT applications. For instance, in smart large buildings and campuses, local information processing will be very likely required, hence requiring spatial decomposition of the LLN.

## 8. Conclusions and Future Work

In this paper, we propose a new variant of the RPL protocol that is suitable for Agricultural Low power and Lossy Networks. This protocol is intended to be used in a farmland with several parcels (or partitions). We called this protocol PA-RPL, standing for Partition Aware-RPL. This protocol ensures the creation of a tree-like structure, DODAG, where nodes located in a given parcel are gathered in a same sub-DODAG. We showed that when only few parcels are simultaneously monitored, the proposed algorithm performs high gain in energy, reaching up to 20%. Moreover, our results show that when parcel level in-network data aggregation is performed, energy saving substantially improves, up to 40%.

PA-RPL is expected to induce longer paths. Consequently, one may anticipate higher latency. However, it is important to note that PA-RPL intrinsically reduces the number of channel contentions, which has a positive impact on the latency. A similar balance can be seen when considering the energy: longer paths are expected to induce higher energy consumption, while our PA-RPL helped gain up to 40% of energy consumption compared to RPL. On the other hand, in the context of our considered application, latency is not a very critical parameter: In all our considered scenarios, the data-sending period is set to at least 5 s, representing at least one order of magnitude higher than the expected end-to-end delay. Nevertheless, a deeper investigation of the impact of PA-RPL on latency will conducted as future work.

Additionally, in this paper, the performance assessment of our proposal was solely made against RPL. This is justified by the fact that our proposal, PA-RPL, is an extension of RPL. Considering other protocols (Hierarchical, cluster-based, etc.) in the comparison is left as a future work, and would be beneficial and worth investigating. However, this would need further adaptation of these protocols to make the clusters/hierarchy reflect the geographical partition of the farmland into parcels. In fact, many of the hierarchical protocols that are already studied in agricultural applications (such as LEACH and APTEEN) are rather applied in different contexts (not used in the IoT context).

Finally, the performance evaluation was limited to the simulation of different scenarios. To effectively measure the efficiency and robustness of our proposal in terms of the defined measures and metrics, we plan considering validation tests in real setup and in different situations. A testbed is currently under development and shall be used in future research.
